# Monitoring and assessment of cadmium, lead, zinc and copper concentrations in arable roadside soils in terms of different traffic conditions

**DOI:** 10.1007/s10661-020-8120-x

**Published:** 2020-02-01

**Authors:** Artur Szwalec, Paweł Mundała, Renata Kędzior, Justyna Pawlik

**Affiliations:** Departement of Ecology, Climatology and Air Protection, University of Agriculture in Krakow, Mickiewicz Av. 24/28, 30-059 Krakow, Poland

**Keywords:** Arable topsoil monitoring, Heavy metals, Traffic emissions, Secondary roads

## Abstract

Quantification of the contributions from traffic source to arable roadside soil heavy metal loadings is a challenge. The contribution depends on: traffic intensity, road type and distance from the road. At a field scale (3.9 ha), 720 topsoil samples were taken. The aim of the study was to monitor and assess the impact of regional/local roads with various conditions of traffic and period of use on the distribution of Cd, Zn, Pb and Cu in the arable roadside topsoil in their vicinity. PCA indicated the occurrence of two main gradients of 11 environmental elements influencing the distribution of heavy metals in the soils of the neighbouring land. The first gradient was associated mainly with the distance from the edge of the road. The second gradient was associated with the degree of contamination of the soils and with the road type, defined by the traffic volume and period of being use. Anova reviled lack of influence of the factors for Cu contents. Unlike Cu, for Cd, Pb and Zn, the significant impact was observed for both factors and interactions between them. The concentrations of Cd, Pb and Zn, regardless of the distance from the road were 0.21–0.58 mg Cd kg^−1^ d.m., 13.60–41.96 mg Pb kg^−1^ d.m. and 40.31–63.97 mg Zn kg^−1^ d.m. In case of increasing distance from the road, generally the contents of Pb, Zn and Cd contents were decreasing. However, only in the case the oldest and carrying the highest traffic road was a clear, statistically significant differences noted for following distances from the road on the content of Cd, Pb and Zn. Analysis of spread gave trend curves, for Pb, Cd and Zn they were parabolas. The curves let reduce sapling distances to 65 m, 45 and 47 m for Cd, Pb and Zn, respectively.

## Introduction

The turn of the twenty-first century seems to be a time of motorization, best suited to the freedom, individuality and entrepreneurship of contemporary human beings. In recent decades, petroleum consumption (petrol or diesel fuel) has been growing much faster in transport than in other sectors. This is due to individuals’ growing need to move from place to place (Chan et al. 2010). In India, for example, combined consumption of petroleum and diesel fuel in 1980–2000 increased four times, and road transport was the largest consumer of liquid fuels (nearly 35% combined). Mobility of European Union residents has been growing as well, generating a substantial increase in the number of cars on the roads. According to Schafer and Victor (2000), the total mobility of the world’s citizens at the turn of the millennium was 23 billion km, and will reach 105 billion km in 2039. Urbanization and industrialisation are the parallel processes increasing transport of cargo and people. Pearl River Delta (south of China) is an example. Farms, rural villages and cultivated soils of the Delta have been change in one of the most densely urbanized region in the world with in last 30 years (Hu et al. 2013). A natural consequence is the construction of motorways in China (Yu et al. 2016); roads in India (Pradhan and Bagchi 2013); in Eastern and Central Europe; and spectacular bridges, viaducts, tunnels, motorway junctions and ring roads all around the world. Due to principle, motorways are avoiding: forests, national parks, nature reserves, areas of Natura 2000, villages, towns and cities. So the farmland is the space preferably chosen for motorways placement. Unfortunately, motorways can have negative effects as well (Quinche and Curzydło 1972; Forman and Alexander 1998). One of them is contamination. Unlike the other, traffic emissions will have been growing in global scale (Pucher et al. 2007).

The effect of road transport on the environment, and thus on roadside soil ecosystems is currently vastly more significant than that of any other transport sector. What is more motor vehicle emissions are currently one of the main sources of pollution of the natural environment. Current standards include construction and operation of low-emission vehicles and improvement of traffic flow (building ring roads, viaducts and grade-separated junctions). Obviously, the impact of large motorways must be studied (Werkenthin et al. 2014; Garcia and Millán 1998; Xu et al. 2014; Isen et al. 2013), but we must not forget smaller roads having a regional or even local impact. These nearly always cut through agricultural land and are usually less well protected from the effects of emissions. There are heavy metals (Cd, Pb, Zn and Cu) with cumulative coefficients of 10–600 among the substances emitted form traffic (Xu et al. 2014). The meaning of those global contaminants is confirmed by the fact that their permissible contents are enshrined in the Regulation of European Commission 1881/2006 on Food Quality with two amendments 488/2014 and 1005/2015 for Cd and Pb respectively.

In Poland, global motorization process takes on a local character, in which the negative impact of roads and motorways is intensified. In the second decade of the twenty-first century, automotive emissions are one of the main sources of pollution of the natural environment in Poland. This is directly linked to the significant increase (over 316% in 1990–2018) in the number of motor vehicles on the country’s roads. Old vehicles constitute the highest percentage. Cars aged 0 to 5 years account for 9.6%, buses 8.8, lorries 11.1% and tractors 30.5%. The remaining vehicles are more than 5 years old. This group is dominated by 10–15-year-old vehicles, often heavily used (PCSO 2018). At the same time, the number of motorways, express roads and parking places is increasing much more slowly than the number of vehicles. In traffic monitoring and assessment studies, a lot of attention is dedicated to urban areas (Akbar et al. 2006; Grigalavičiené et al. 2005; Van Bohemen and Van de Laak 2003), much less to farmlands in neighbourhood of busy motorway. Very little attention is paid to grassland and arable land located close to national or regional roads, and which is usually much less well protected against the emissions. There are questions whether the impact of the regional road is important for the fertility and environmental quality of soil ecosystems. And if so, what is the range and intensity of this influence? How can it be mathematically calculated? What are the environmental factors created by neighbourhood of a road? How do the factors influence the soil ecosystems? How can be the spread of pollutants be monitored?

The aim of the study was to monitor and assess the impact of regional/local roads with various conditions of traffic and period of use on the distribution of Cd, Zn, Pb and Cu in the arable roadside topsoil in their vicinity. It was hypothesised by the period of use, traffic volume and increasing distance from the road affect the contents of heavy metals in the arable surface layer (0–0.2 m) of the soil. Assessment was also made to identify the heavy metal contamination levels. A monitoring system was suggested and discussed.

## Material and methods

### Selected roads and farmlands, a monitored area

Transport routes of varying age and traffic volume, located in a rural area in the Jędrzejów region (southern Poland), were selected for the study. The first of these was express road S7 (Jędrzejów-Kielce section—JK), connecting Rabka Zdrój, Krakow, Kielce, Warsaw, Elbląg and Gdańsk, which has been in use for 50 years and has a mean traffic volume of 20,000 vehicles per day. The second selected road was the Eastern Jędrzejów Ring road (EJR), which was added to S7 in order to reduce traffic in the town. At the time of the study, it had been in use for 10 years and the traffic volume was 18,000 vehicles per day. The third road was the Northern Jędrzejów Ring Road (NJR), newly constructed and in use for 1 year, with traffic volume of 10,000 vehicles per day. The NJR road was added to national road DK78, connecting two Polish regions: Upper Silesia and the Świętokrzyskie Voivodeship. NJR, JK and EJR meet north of Jędrzejów (GPR 2015). In the area studied, these roads cut through agricultural land. Agriculture in the area is traditional, without large specialized and intensively run farms, but the volume of agricultural production goes beyond the local scale and reaches a regional one. There are also no significant industrial plants in the area.

### Soil monitoring

**Fig. 1:**
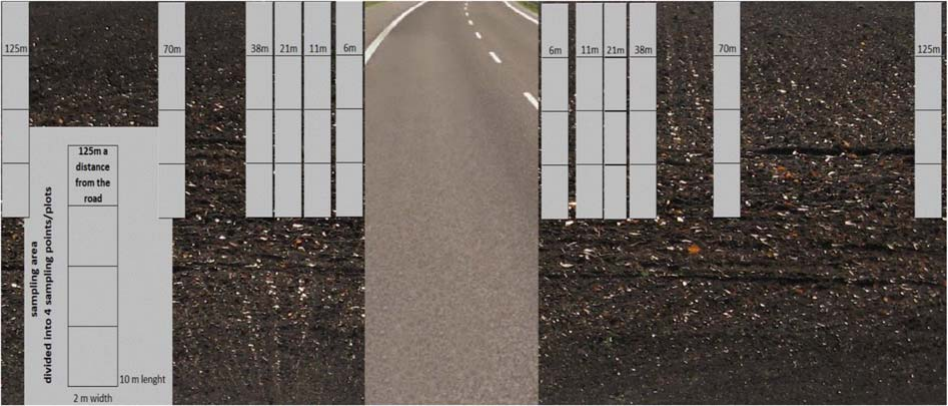
Sketch of the segment: sampling areas, research points/plots, distance from the road, number of repetition point/plots and the size of the point/plots. Five segments make the section, the monitoring system of the road

Five segments of each of the analysed sections of road, i.e. NJR, EJR and JK, were designated for analysis. Soil samples were collected in study areas located in these sections, distributed symmetrically on each side of the road at distances of 6, 11, 21, 38, 70 and 125 m from the edge of the roadway (Fig. 1).

Distances of less than 5 m were omitted due to transformation of the soil and potential contamination associated with the materials and machines used in building the road. All examined soils were arable. One study area (for instance distance 11 m) consisted of four sampling points/plots, arranged in a row parallel to the road. Each sampling point/plot was a rectangle of about 20 m^2^ (analysed distance + 1 m × 10 m). From each, such rectangle five primary samples were collected from the topsoil, i.e. from a depth of 0–0.2 m, using a soil sampler. Following homogenisation, these constituted an averaged sample with a combined weight of about 600 g. When the material had been suitably prepared (dried, ground and sieved), wet mineralisation was performed using a mixture of concentrated HNO_3_ and HClO_4_ (Ostrowska et al. 1991; Carter and Gregorich 2019). Concentrations of Cd, Pb, Zn and Cu were determined by an atomic flame absorption spectrometry, using a Unicam Solaar M6 spectrometer. In order to check the quality of calibration of atomic absorption spectrophotometer references, materials were used. They were trace metals–clay and trace metals–sandy loam 7 provided by Merck. Recovery were 95% for Cd, 94% for Cu, 97% for Pb and 98% for Zn. In addition, the pH of the samples was determined in a solution of distilled water and KCl by the potentiometric method, content of organic matter by annealing and granulometric composition by the Casagrande method as modified by Prószyński (Ostrowska et al. 1991).

### Statistical analyses and samples repeatability

The principal component analysis was used to evaluate significant environmental factors associated with the concentrations of heavy metals in the soil at the sampling points. The Shapiro–Wilk test was used to determine distributions of dependent variables. As the distributions had characteristics of a normal distribution, analysis of variance (two-way with repeated measures) and spread analysis was applied, using the Statistica for Windows v. 12.0 (StatSoft 12). The statistical analysis was performed using the CANOCO v.4.5 statistical package (Braak and Smilaner 2012). There were 720 soil samples collected. Each soil sample was mineralised twice independently from each other. All samples were collected at the same year. Typical for agronomic science, a multiyear experiment was deliberately omitted. It was due to very low year to year variability of Cd, Pb, Zn or Cu contents in soil. It is a typical procedure in heavy metal monitoring and assessment in soil (Zhang et al. 2012; Wu et al. 2011; Nabulo et al. 2006; Carrero et al. 2013; Jankaitė et al. 2008; Tóth et al. 2016).

## Results and discussion

### Heavy metal, silt, organic matter contents and pH

The highest variation (coefficient of variation, *v* = 278%) was noted for Cu content (Table 1). This resulted from the relatively large number of results indicating a very low concentration, i.e. on the threshold of deficiency of this element in the soil (Kabata-Pendias 2011). Content below 3 mg Cu kg^−1^ d.m. was noted in nearly 7% of the results and less than 7.1 mg Cu kg^−1^ d.m. in nearly 51% of results, while content above 21 mg Cu kg^−1^ d.m. was observed in only 4% of results. There was no Cu measured as a traffic roadside soil pollution at the beginning of a road impact studies Lagerwerff and Specht (1970), Quinche and Curzydło (1972) and even letter Garcia and Millán (1998). However, in many latest studies, Cu has been identified as traffic pollution; for this reason, Cu was included in our research. Copper emission is being caused by wearing of brake pads in road vehicles (Straffelini et al. 2015; Wahid 2018). The chosen roads and two exits to Jędrzejów town have all grade-separated junctions. There are also no any zebra crossings: public footpaths, bike paths or bridle paths. Normally, the drives do not need to brake the vehicles, so there is no copper emission. The emission appears only in case of emergency braking in case of a bump or accident. The highest Cu content, i.e. 226.2 mg Cu kg^−1^ d.m. was noted at a distance of 6 m from the edge of EJR road, appears to be random and may be the result of local contamination with pesticides containing this metal or road incident, and does not appear to be linked to the direct impact of the factors under consideration.

**Table 1:** Heavy metal, pH silt and organic matter contents according to researched roads

Metal/statistic	Cd	Pb	Zn	Cu	pH		Silt	Organic matter
	mg kg^−1^ d.m.				H_2_O	KCl	%	%
Road	NJR							
Minimum	0.10	7.26	13.1	1.50	5.5	4.7	6	0.6
Maximum	0.98	51.90	152.9	37.80	7.5	7.3	35	5.5
Arithmetic mean	0.363	21.561	52.98	8.732	6.76	6.35	22.6	2.56
Geometric mean	0.320	19.556	45.60	6.967	6.73	6.31	20.4	2.08
Median	0.350	19.250	50.20	6.450	7.00	6.65	26.0	2.40
Coefficient of variation (%)	47	45	55	77	9	12	39	63
Road	EJR							
Minimum	0.10	6.60	17.3	2.20	4.7	3.8	7	0.5
Maximum	0.46	20.60	88.3	226.20	7.6	7.7	20	1.5
Arithmetic mean	0.214	13.603	40.33	10.230	6.59	6.19	14.4	1.07
Geometric mean	0.198	13.204	36.89	6.063	6.55	6.12	13.9	1.03
Median	0.195	14.100	36.10	5.850	6.65	6.20	14.5	1.10
Coefficient of variation (%)	41	23	45	278	11	14	25	24
Road	JK							
Minimum	0.15	13.80	25.30	5.50	5.9	5.5	8	0.6
Maximum	1.54	101.30	135.40	21.60	7.5	7.3	38	5.6
Arithmetic mean	0.576	41.960	63.970	8.948	6.68	6.34	25.0	3.26
Geometric mean	0.462	35.723	59.472	8.534	6.67	6.35	22.9	2.66
Median	0.465	35.000	59.950	8.400	6.70	6.35	29.0	3.55
Coefficient of variation (%)	66	59	39	34	6	7	37	51

Unlike reports by Zhang et al. (2012), average Cu content for farmland roadside soils was about 20 mg kg^−1^ d.m. with low variability (*v* < 39%). High coefficients of variation were also noted for the concentrations of Cd, Pb and organic matter (66%, 59% and 63% respectively). Variation in the content of Zn and silt fractions was moderate (55% and 39%).

### Source of misleading in monitoring and assessment

There are four problems appeared in monitoring and assessment of heavy metals contamination of roadside soils. First of them is the depth of sampling and the second is the distance from the edge of the road. Jankaitė et al. (2008), studying the impact of motorways in Lithuania on the chemistry of adjacent soils, collected samples from a depth of 0–0.10 m. Similarly, in northern France (the A31 motorway), Viard et al. (2004) also analysed soil samples from a depth of 0–0.10 m. The same sampling depth was chosen by De Silva et al. (2016) in Melbourne, Australia. Researchers investigating the same problem in Greece collected samples from a layer of 0–0.01 m (Christoforidis and Stamatis 2009). Following depths were applied: 0–0.05 m by Garcia and Millán (1998) in Gipuzoka region, north of Spain and Wawer et al. (2015) in nine different cities in Europe and in Asia. Wu et al. (2011) sampled 0–0.15 m in Changzhou region east of China. Due to ploughing standards in Poland, i.e. 0–0.2 m depth and commonly used in this country Kabata–Pendias’s et al. method (1993) of assaying heavy metal contents in topsoil (0.0–0.2 m), the sampling depth had to be 0–0.2 m in the present study. An advantage with arable land in the vicinity of roads is the existence of the soil ploughing layer; tillage on soil causes the dilution of the deposited metals. According to Werkenthin et al. (2014), the depth of soil sampling is crucial for the determined heavy metal content. Each increase of the sampling depth (up to 20 cm) was reducing the content of metals, as well as reduced the differences in content at individual rising distances. The second problem is distance, i.e. the method of measuring the distance from the road. There seems no to be one benchmark to start the measurement. Yan et al. (2012) in Nepal were sampling at 0 m from the road edge. Carrero et al. (2013) were sampling 0.5 m (west side) or 3 m (east side) from the road in condition of Bilbao region (North of Spain). De Silva et al. (2016), near Melbourne in Australia, manage to collect samples 2 to 5 m from the road edge. According to Werkenthin et al. (2014), a “road edge” is the end of asphalt or concrete belt. Soils 0–5 m are named constructed soil, 5–10 m disturbed soil and just above 10-m undisturbed soil. It was assumed in the present research work, in referring to Quinche and Curzydło (1972), Curzydło (1989), that the influence of distance is a conical curve (parabola or hyperbole). Because of this, soil samples were collected more frequent in closers distances (6, 11, 21, 38 m). The disturbed soil was also taken into consideration (the first sampling distance is 6 + 1 m). The further distances were 70 m and 125 m. After logarithmic transformation of the numbers of distances, a straight line is created. Finally, the assessment depends on the place, where the soil is located and an assaying method. According to Zhao and Li (2013), there are soils in following places: central urban, central suburban county, rural town, rural village areas and urban village areas. Only rural village areas were chosen in the present study. There are four methods heavy metals assessment in the soil: direct comparison, geoaccumulation index, potential ecological risk index and Nemerow pollution index (Zhao and Li 2013). The direct comparison method was applied in the present study.

### Level of contamination and assessment

The soils should be regarded as 98.34% uncontaminated (sum of degrees 0^o^ and I^o^) with Cd, 98.9% uncontaminated with Pb, 99.40% with Zn and 98.90% with Cu. For a few isolated samples (0.60% to 1.67%), the soil was slightly contaminated with Cd, Pb and Zn; while in the case of Cu (1.10%), heavy contamination was noted in the problematic sampling area discussed above (Table 2) (Kabata-Pendias et al. 1993).

**Table 2:** Estimation of heavy metal contents in researched topsoils irrespective of localisation (i.e. distance and road type) according to a Kabata–Pendias’s et al. (1993) method

Metal/degree	Level name	Cd	Pb	Zn	Cu
		%			
0	Natural/background	82.77	90.60	79.40	97.80
I	Increased concentration	15.56	8.30	20.00	1.10
II	Slight contamination	1.67	1.10	0.60	0.00
III	Medium contamination	0.00	0.00	0.00	0.00
IV	Strong contamination	0.00	0.00	0.00	1.10
V	Very strong contamination	0.00	0.00	0.00	0.00

The Kabata–Pendias et al. method gives agricultural recommendations for each level. There is no prohibition in crops possible to be cultivated in natural content level. In increased concentration level, there is one limitation—vegetables for infants are prohibited. Over 98% of estimated soil belong to sum of 0 and 1 levels, i.e. areas with no limitation if food production for adults (Table 2).

**Fig. 2:**
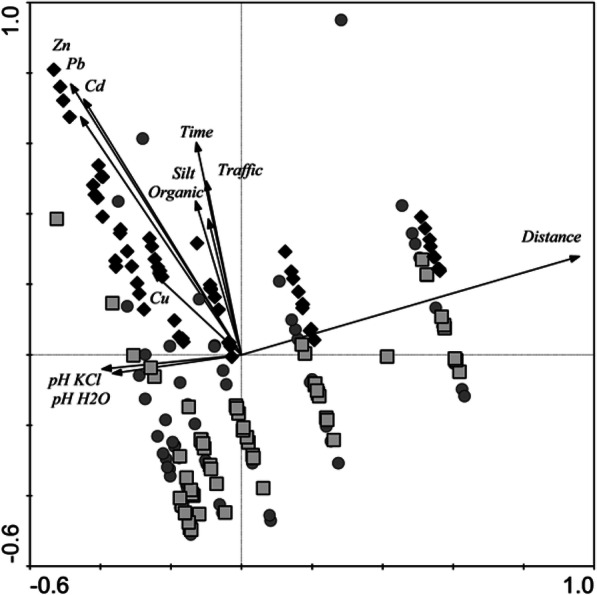
Results of principal component analysis performed on 11 environmental characteristics of localities along three road’s type (square, EJR road; circle, NJR road; diamond, JK road)

The principal component analysis, based on 11 environmental parameters, revealed two main gradients of environmental (Table 3, Fig. 2) conditions among the study areas, along which the sampling points were arranged. The first gradient was associated with a strong influence of the distance from the road on the distribution of heavy metals in the soils. The first axis, describing 47% of the variance in environmental characteristics, was positively correlated mainly with the distance from the road and negatively with soil pH (both in KCl and H_2_O) (Fig. 2.). The second gradient was associated with the type of road, i.e. how long it had been used and its traffic volume, which was also reflected in the concentrations of heavy metals in the soils. The second axis, describing 25% of the variance, was positively correlated with soil quality parameters: content of heavy metals (Zn, Pb and Cd), silt fractions (grain size < 0.02 mm), and organic matter, and with parameters characterizing the road: traffic volume and the time of use. The third ordination axis, describing 16% of the variance, is positively correlated with Cu content in the soils, while the fourth axis, describing 7% of the variance, was inversely correlated with ordination axis AX2 in terms of the traffic volume and time of use (Fig. 2, Table 3).

**Table 3:** The loadings of environmental parameters to the first four axes of PCA

Environmental variables	AX1	AX2	AX3	AX4
Distance—distance to the road (m)	*0.9581*	0.279	0.0564	0.0294
pH H_2_O	*0.3662*	0.0533	0.0522	0.0761
pH KCl	*0.3953*	0.0406	0.0667	0.0352
Cd (mg kg^−1^ d.m.)	0.4562	*0.6752*	0.2653	0.1425
Zn (mg kg^−1^ d.m.)	0.484	*0.7686*	0.0903	0.3738
Pb (mg kg^−1^ d.m.)	0.4471	*0.7256*	0.3178	0.0107
Silt fraction below 0.02 mm	0.1296	*0.4365*	0.1233	0.0253
Organic-organic matter (%)	0.0937	*0.3854*	0.2009	0.0684
Traffic (1000 cars day^−1^)	0.1005	*0.6929*	0.3422	*0.6978*
Time-road’s lifetime (year)	0.128	*0.653*	0.415	*0.6487*
Cu (mg kg^−1^ d.m.)	0.2513	0.233	*0.9007*	0.2616

The italic means the values with the highest variation percentage.

The results of the principal component analysis clearly indicate that the distance from the road and type of road (defined as traffic volume and time of use) were factors affecting the distribution of heavy metal concentrations in the soil. This was confirmed by the results of two-way analysis of variance (road type, distance, type-distance interaction). The two-way analysis of variance for these parameters showed statistically significant differences in the content of Cd (*F* = 10.75, *p* < 0.0001), Pb (*F* = 18.01, *p* < 0.0001) and Zn (*F* = 4.21, *p* < 0.0001). *F*-table value for the experiment was 1.89. Only in the case of silt fractions (*F* = 0.01, *p* = 1.00) and Cu (F = 1.08, *p* = 0.377) there were no significant differences observed. In this case, Cu is a normal component of the soil. In case of increased concentration coming from agricultural sources like inappropriate mineral fertilizing or from manure from inappropriate fed (supplemented with Cu) livestock, this could explain a different pattern of Cu distribution in the researched topsoil. Nevertheless, it should be noted that contamination of the environment with this metal also had a source in the use of vehicles (e.g. wear on brake linings; secondary emissions) (Aslam et al. 2013; Adachi and Tainosho 2004; Lu et al. 2009).

The type of road, i.e. its traffic volume and time of use, was found to influence the content of Cd, Pb and Zn (irrespective of distance) (Table 4.). The distance from the road (irrespective of its type) also affected the content of these metals. For all roads, the concentrations of Cd in topsoil were decreasing 0.49 mg kg^−1^ d.m. > 0.45 = 0.43 > 0.38 > 0.30 > 0.27 mg kg^−1^ d.m., with RSD = 0.027 respectively for 6 m, 11, 21, 38, 70 and 125 m. The same trend was noticed for Pb 34.14 > 30.71 > 26.40 > 22.67 = 21.67 > 18.64 mg kg^−1^ d.m. with RSD = 1.487 for the same distances in increased ordered. Finally, for Zn, the tendency was 65.24 > 59.55 > 51.94 = 50.34 > 42.14 > 45.36 mg kg^−1^ d.m. The interaction of these characteristics is statistically significant as well. On the other hand, neither road type nor distance, nor the interaction of these characteristics, were significant for Cu content. The highest concentrations of Cd, Pb and Zn were determined in the soils adjacent to the JK road, which had been in use for the longest period − 50 years. An interesting phenomenon is the higher content of Cd, Pb and Zn in the soils near the NJR road, which had been in use for a year and had lighter motor vehicle traffic than in the soils near EJR road. It should be noted that these soils generally had a lower (0^o^–1^o^) degree of contamination with these metals. Soil samples collected near EJR road had lower mean content of silt (grain size < 0.02 mm), at 14.4% as compared to 22.6% for the NJR road soils (Table 4). The strong positive correlation between the content of the silt fraction and the content of heavy metals in the uncontaminated soils (Kabata-Pendias 2011) suggests that the relationship identified at the current period of use of these roads (1 and 10 years respectively) is probably due to the higher natural biogeochemical background of the soils in the vicinity of NJR road as compared to EJR road, and not to the effect of the road emission. Analysis of the effect of the distance from the road on the content of the heavy metals tested in the neighbouring soils reveals that it decreases with increasing distance from the edge of the road (irrespective of the road type). The highest mean concentrations of Cd (0.49 mg kg^−1^ d.m.), Pb (34.14 mg kg^−1^ d.m.) and Zn (65.24 mg kg^−1^ d.m.) were noted in the samples collected at a distance of 6 m from the road. The lowest content of these elements was noted at a distance of 125 m from the road, i.e. 0.27 mg Cd kg^−1^ d.m., 18.64 mg Pb kg^−1^ d.m. and 45.36 Zn mg kg^−1^ d.m. The mean content of Zn at a distance of 70 m, i.e. 45.36 Zn mg kg^−1^ d.m., did not differ significantly from the content at 125 m (Table 5). Decreasing of heavy metal content as a function of increasing distance from the road has been typically observed and reported (Lagerwerff and Specht 1970; Hjortenkrans et al. 2006; Curzydło 1989). An exception to this rule is roadside farmland soil located near Trishuli Highway in Nepal. However, the researched soils are located in mountain, 800–1800 m above sea level and this could be the most important factor (Zhang et al. 2012; Yan et al. 2012).

**Table 4:** Comparison of mean contents of Cd, Pb, Zn, Cu and the silt fractions in the soils adjacent to the analysed road sections, irrespective of distances;

Road/Metal	Unit	NJR	EJR	JK	RSD
Cd	mg kg^−1^ d.m.	0.36	0.21	0.58	0.05
Pb		21.56	13.61	41.96	2.56
Zn		52.98	40.34	63.97	5.32
Cu		8.73a	10.23a	8.95a	No differences
Silt content	%	22.57a	14.40	24.97a	2.805

Lowercase letters a, b and c in rows designate statistically non-significantly different values, Tukey’s test, reasonable significant difference (RSD)

**Table 5:** Mean concentrations of Cd, Pb and Zn for individual roads and distances (significances for interaction of type of road and distance)

5.1.	[m]	NJR	EJR	JK
		mg kg^−1^ d.m.		
		Cd		
	6	0.26^a^ aijkl	0.15^g^ ghl	1.06^-^
	11	0.36^b^ bcdefk	0.20^h^ aghijl	0.79^-^
	21	0.43^c^ bcdef	0.25^i^ ahijkl	0.60^-^
	38	0.41^d^ bcdef	0.25^i^ ahijkl	0.47 cd
	70	0.34^e^ bdefk	0.24^j^ ahijl	0.32^k^ abdefik
	125	0.39^f^ bcdefk	0.20^h^ ahijkl	0.22^l^ aghijkl
	RSD	0.07		
5.2.	[m]	NJR	EJR	JK
		mg kg^−1^ d.m.		
		Pb		
	6	14.58^a^ ahijkl	10.67^g^ ghjl	77.17^-^
	11	21.29^b^ bcdefmn	13.12^h^ aghikl	57.72^-^
	21	23.61^c^ bcdefm	14.41^i^ ahikl	41.20^-^
	38	22.26^d^ bcdefmn	14.28^j^ aghijkl	31.48^-^
	70	24.90^e^ bcdefm	15.44^k^ ahikl	24.67^m^ bcdefm
	125	22.73^f^ bcdefmn	13.66^l^ aghijkl	19.52^n^ bdfn
	RSD	3.64		
5.3.	[m]	NJR	EJR	JK
		mg kg^−1^ d.m		
		Zn		
	6	50.08^a^ abcefghmn	44.02^g^ aeghklno	101.61^-^
	11	52.72^b^ abcdfm	44.27^h^ aeghklno	81.66^-^
	21	52.99^c^ abcdfm	38.09^i^ eghiklno	64.74^-^ df
	38	60.25^d^ bcdfm	37.24^j^ eghiklno	53.52^m^ abcdfm
	70	44.36^e^ aeghijklno	37.81^k^ eghijklno	44.24^n^ aeghijklno
	125	57.45^f^ abcdfmn	40.58^l^ eghijklno	38.05^o^ eghijklno
	RSD	7.56		

Lowercase letters in superscripts are assigned to particular data. Lowercase letters in rows are designating statistically non-significant values, ‘-’ there is no need for a letter

Analysis of the effect of road type and distance clearly indicated that the JK road, with the longest period of use (50 years) and the highest traffic volume (20,000 vehicles per day) was a source of contamination of the soil with Cd, Pb and Zn. A statistically significant decrease is noted in Cd content at each successive distance, from 1.06 mg kg^−1^ d.m. for a distance of 6 m to 0.22 mg kg^−1^ d.m. for a distance of 125 m. The same relationship for this road was observed in the case of Pb; its content was highest closest to the road, at 77.17 mg kg^−1^ d.m. and lowest at the furthest distance at 19.52 mg kg^−1^ d.m. Similarly, Zn content decreased from 101.61 to 38.05 mg kg^−1^ d.m., while the concentrations of this metal at distances of 70 and 125 m do not differ significantly (Table 5). This slightly different pattern in the decrease in Zn content as compared to the concentrations of Cd and Pb could be due to the fact that Zn is a physiological element, and concentrations of 64.74 to 38.05 mg kg^−1^ d.m. corresponding to the range of its typical physiological content in soil (Kabata-Pendias 2011). The lack of statistically significant differences in the interaction of these factors for EJR and NJR roads in the concentrations of Cd, Pb and Zn could be linked to the relatively short (in comparison with JK) period of use of these roads, and thus the lack of a cumulative effect of these elements in the topsoil. It should be noted that leaded petrol was withdrawn from the market in Poland in March 2005, and the use of both of these roads began after this time. In addition, relatively good traffic flow is ensured on both of these ring roads and this of course reduces fuel consumption, and thus emissions, which are also influenced by pro-environmental technologies implemented in new vehicles (Aslam et al. 2013). Apart from the factors analysed in the study, the distribution of the metals tested in the soils adjacent to the transport routes may also be influenced by natural factors. De Silva et al. (2016) lists wind, rain and air movement caused by gravity on sloping terrain, Curzydło (1989) adds vegetation. In the present study arable soils were selected because they are dominant in the crop structure of the area. A lowland (flat) area was chosen, without trees or shrubs along the road. Both sides of the road were studied (symmetrically): the east and west sides in the case of JK road and EJR road and the north and south sides in the case of NJR road. Two-way analysis of variance (road type and side of the road) did not show a significant effect of the side of the road on the content of the metals tested. The western and south-western winds dominant in this area had no significant effect on the distribution of metals. A high 57% percentage of calm conditions is characteristic of the area studied (JDEP 2009).

**Fig. 3:**
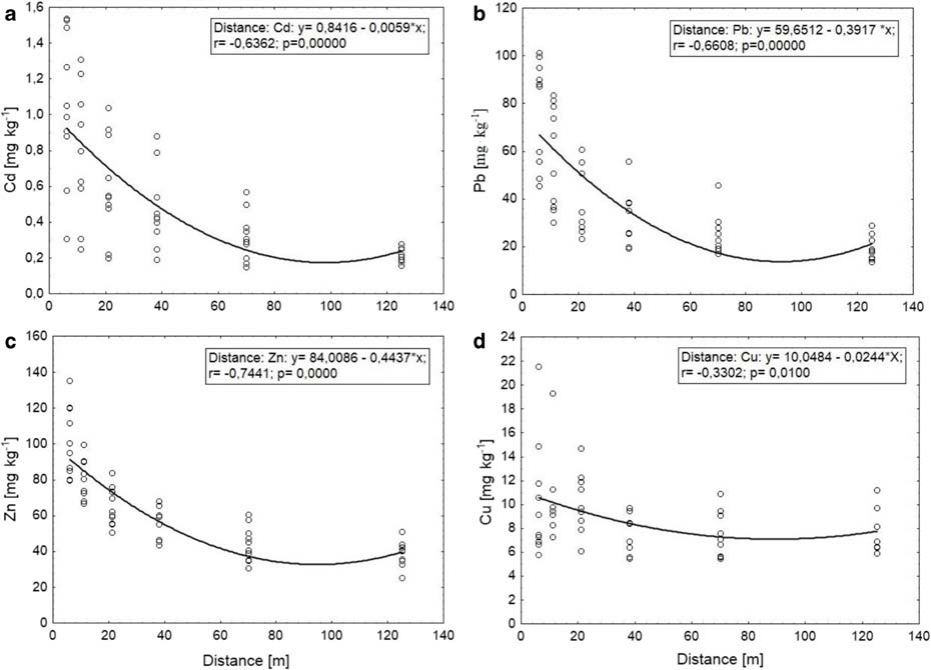
**a**, **b**, **c**, **d** Spread analysis of Cd, Pb, Zn and Cu concentration in JK road topsoil

Analysis of the lack of significant differences between Cd, Pb and Zn concentrations (Table 5) makes it possible to restrict the number of roads analysed for spread analysis to JK road. The trend curves are parabolas (with a domain of [6125 m]) *y*_Cd_ = 8.8919 × 10^−5^ × *x*^2^– 0.0174*x* + 1.0293 (Fig. 3a), *y*_Pb_ = 0.0071*x* ^2^–1.3132*x* + 74.612 (Fig. 3b), *y*_Zn_ = 0.0075 *x* ^2^–1.418*x* + 99.8272 (Fig. 3c), and *y*_Cu_ = 0.0005*x* ^2^–0.09*x* + 11.1142 (Fig. 3d). The correlation coefficients between estimated curve and the data are *r*_Cd_ = −0.6362, *r*_Pb_ = −0.6608, *r*_Zn_ = − 0.7441 and *r*_Cu_ = − 0.3302 (Fig. 3a–d respectively). Unfortunately, equations for the relationship between distance and other factors affecting topsoil pollution adjacent to the roads are very rare (Yan et al. 2012; Swaileh et al. 2004; Wu et al. 2011; Nabulo et al. 2006; Grigalavičiené et al. 2005). Unlike us researches form Lithuania, who were assaying impact of Vilnius–Kaunas–Klaipeda motorway on forest soil proposed a logarithmic function *y* = − *a* × lnx + b, and had higher correlation coefficients *r*_Pb_ = 0,89 and *r*_Cd_ = 0,80 (Grigalavičiené et al. 2005). The equations have a large practical application. The equations in similar conditions can be changed. As a “y” value put down a permissible topsoil metal content and count the distance from each and above the metal content is equal or lower than permissible.

In Poland, to assay the content of heavy metals in topsoil, the Kabata–Pendias et al. method (1993) is being used for both scientific and practical purposes. For light soils, the following content limits are permissible: 0.3 mg kg^−1^ d.m. for Cd, 30 mg kg^−1^ d.m. for Pb and for Zn 50 mg kg^−1^ d.m. After substituting for the inequality, the distance for a road like JK is not less than 65 m, 45 and 47 m for Cd, Pb and Zn, respectively.

What are the reasons that the state of art in case of the chosen roadside topsoil is go good? There are three reasons. First of them is vegetation. Traffic pollution is emitted to the air and transported to the air. The vegetation is a barrier, a biofilter between the air and the soil. Growing season near Jędrzejów is about 220 day. It is about 60% of the year. In this area, like in the whole Poland, winter cereals are the most popular crops, so the period of vegetation protecting the soil is being increased. Finally, the most of the fitomass (crops) are being taken from these particular areas. The second reason is the dilution process. The contaminant in this case of heavy metals reach the soil “needs” to pollute the both sides of road up to the distances of 65 m (for Cd), 45 m (for Pb) and 47 m (for Zn). Finally, there is dilution by ploughing. This agrotechnique measure is very effective in slowing down the soil pollution process. The third reason is connected with time flow. The vehicles 50 years ago were completely different than now. The traffic density has been dramatically changed. A prohibition on selling leaded gasoline stopped roadside topsoil pollution.

The last but not least is the modernization of discussed roads junction. The fluent vehicle movement reduces the traffic emission.

## Conclusions

We compared and analysed Cd, Cu, Pb and Zn contents in roadside arable topsoils. The principal component analysis indicated the occurrence of two main gradients elements influencing the distribution of heavy metals in the topsoils. The first gradient was associated mainly with the distance from the edge of the road. The second gradient was associated with the degree of contamination of the soils and with the road type, defined by the traffic volume and time of use. The analysis of variance indicates influence of type of road on Cd, Pb and Zn contents in topsoil. There was no influence on Cu contents. The highest concentrations of Pb, Zn and Cd were in topsoil of JK (50 years of use, 20,000 vehicles per day), the lowest for EJR road (10 years and 18,000 vehicles per day), and the medium for NJR road (1 year and 10,000 vehicles per day). Also, interaction between distance from the road and road type were statistically important. The most clear influence of increasing distance from the road on Pb, Cd and Zn contents appeared in case of JK road. The concentrations of Cd, Pb and Zn were decreasing. In case of interactions stated as statistically significant, the analysis of spread was carried on. Curves for heavy metal contents for the JK road topsoil were parabolas: *y*_Cd_ = 8.8919 × 10^−5^ × ^2^–0.0174*x* + 1.0293; *y*_Pb_ = 0.0071*x*^2^–1.3132*x* + 74.612; and *y*_Zn_ = 0.0075*x*^2^–1.418*x* + 99.8272. Coefficients of correlations between data and curve equations were *r* = − 0.6362 (Cd), *r* = − 0.6608 (Pb), and *r* = − 0.7441 (Zn). This allowed to determine the minimum distance from the road to the limit where the Cd, Pb and Zn concentrations are acceptable or lower. The distances are 65 m, 45 and 47 m respectively for Cd, Pb and Zn. Finally, according to the Kabata–Pendias method, only 1.67% of samples were slightly contaminated with Cd, 1.10% with Pb, 0.60% with Zn and 1.10% moderate contaminated with Cu.
